# Neuro-Mechanical Regulation of Vascular Smooth Muscle Cell Behaviour Under Ageing-Associated Substrate Stiffness

**DOI:** 10.3390/cimb48080823

**Published:** 2026-08-12

**Authors:** Yumin Hou, Sejal Singal, Pamela Swiatlowska, Jose L. Sanchez-Alonso

**Affiliations:** National Heart and Lung Institute, Faculty of Medicine, Imperial College London, London W12 0NN, UK; yuminhou1205@outlook.com (Y.H.); s.singal22@imperial.ac.uk (S.S.)

**Keywords:** vascular smooth muscle cells, sympathetic neurons, substrate stiffness, mechanobiology, vascular ageing, neurovascular interaction

## Abstract

Cardiovascular diseases (CVDs) remain a leading cause of mortality worldwide, and ageing is strongly associated with progressive arterial stiffening. Age-related alterations in extracellular matrix (ECM) mechanics influence vascular smooth muscle cell (VSMC) behaviour, while sympathetic innervation represents an additional regulator of vascular homeostasis. However, how neural signalling interacts with ageing-associated mechanical conditions to regulate VSMC behaviour remains unclear. In this study, an in vitro sympathetic neuron–VSMC co-culture model was established to investigate neuro-mechanical regulation. Primary rat sympathetic neurons and A7r5 VSMCs were cultured on glass or polydimethylsiloxane (PDMS) substrates with defined stiffness (20 and 130 kPa), representing healthy and ageing-associated stiffened arterial environments, respectively. VSMC behaviour was assessed through analysis of cell area, proliferation, migration, cellular Young’s modulus (YM), and DNA damage marker γH2AX. Sympathetic neuronal co-culture was associated with reduced VSMC spreading and decreased γH2AX levels. Under the conditions tested, neural signalling exerted limited effects on cell proliferation and migration. In contrast, increased substrate stiffness promoted cell proliferation and elevated YM. Both neuronal input and substrate stiffness were associated with increased cellular YM. Together, these findings indicate that neural and mechanical cues may jointly influence VSMC behaviour within ageing-associated mechanical environments. This co-culture system provides a controllable platform for studying neuro-mechanical interactions in vascular biology.

## 1. Introduction

The mechanical behaviour of large arteries such as the aorta is governed by multiple factors. These include the composition of the medial layer, where vascular smooth muscle cells (VSMCs) are embedded within an elastin- and collagen-rich extracellular matrix (ECM), as well as biomechanical stimuli such as wall shear stress from intraluminal blood flow, transmural pressure, and cyclic mechanical stretch of the vessel wall [1,2]. Ageing is commonly accompanied by ECM remodelling, including elastin degradation and increased collagen deposition, which together contribute to reduced compliance and a stiffer vascular microenvironment [3,4,5]. These structural changes are closely associated with cardiovascular diseases (CVDs), as increased arterial stiffness and reduced vascular compliance impair vascular buffering capacity, elevate pulse pressure, and promote endothelial dysfunction, collectively driving vascular dysfunction [6,7].

VSMCs are the predominant cell type in the arterial media and are essential for regulating vascular tone and maintaining vessel integrity [8]. A defining feature of VSMCs is phenotypic plasticity, enabling transitions between a contractile phenotype, characterised by high expression of contractile proteins (e.g., α-SMA and SM-MHC) and low proliferation, and a synthetic phenotype associated with increased spreading, proliferation, migration and ECM production [9,10,11,12]. This phenotype switching is tightly coupled to changes in the extracellular environment, including injury- or disease-associated ECM remodelling, and is implicated in vascular remodelling and stiffness progression [11,13,14].

A growing body of mechanobiology research indicates that VSMCs are highly sensitive to the mechanical properties of their microenvironment [15,16]. Through integrin-mediated adhesions, VSMCs sense ECM stiffness and translate mechanical cues into biochemical signalling via focal adhesion assembly and actin cytoskeleton organisation [14,17,18]. Soft matrices within a physiological stiffness range tend to support a more contractile-like state, whereas stiffer substrates promote cell spreading, stress fibre formation and functional features consistent with a synthetic phenotype, together with altered proliferation and migration behaviours [16]. Mechanical cues can also impact nuclear mechanics and DNA damage responses, suggesting a potential link between an increased mechanical load and cellular stress signalling [19].

In addition to mechanical regulation, sympathetic neurons represent an important extrinsic regulator of vascular function and can modulate VSMC behaviour through neurotransmitter release and paracrine signalling [20,21,22]. A growing body of evidence suggests a bidirectional regulatory relationship between neurons and blood vessels: vascular cells can provide trophic support for axons, while sympathetic signalling also participates in regulating VSMC phenotype and vascular remodelling-related processes [20,23,24]. In relation to how ageing affects vascular innervation, Omar and Marshall reported that, in the rat cerebrovascular system, sympathetic nerve fibre density increases during maturation before declining in middle age, while carotid vasoconstrictor responses to norepinephrine also show age-dependent changes, suggesting that ageing may additionally alter vascular responsiveness to sympathetic signalling [25]. More recently, Wagner et al. reported a marked reduction in nerve density in the hearts of aged mice; mechanistically, they found that reduced miR-145 expression in aged vascular endothelial cells relieves suppression of the nerve-repellent factor Semaphorin-3A (Sema3A), thereby promoting local loss of nerve fibres [26]. Together, these findings suggest that ageing may influence arterial innervation; however, this phenomenon appears to be vascular bed-dependent, and whether large arteries, particularly the aorta, undergo reduced innervation density purely as a consequence of ageing remains inconclusive. Even if innervation is altered during ageing, neurovascular communication is unlikely to be completely abolished and may instead undergo remodelling. However, it remains unclear whether the substrate stiffness differences associated with health and ageing further modulate sympathetic neuron–VSMC interactions and jointly influence VSMC behaviour.

In this study, we established a sympathetic neuron–VSMC (A7r5) co-culture on polydimethylsiloxane (PDMS) substrates with defined stiffness to examine how sympathetic signalling and substrate stiffness jointly regulate VSMC behaviour. Atomic force microscopy (AFM) studies have shown that the Young’s modulus of the medial layer in healthy arteries is approximately 20 kPa [27,28,29], whereas ageing or pathologically stiffened vascular tissue, including calcified regions, can reach approximately 130 kPa [27,29,30,31]. Accordingly, two substrate stiffnesses (20 and 130 kPa) were selected to represent physiological-like and ageing-associated stiff mechanical environments, respectively. Conventional glass coverslips were included as an additional control condition. VSMC responses were evaluated using complementary cellular and biophysical readouts, including cell area, proliferation, migration, Young’s modulus (YM), and γH2AX as an indicator of DNA damage response. This study aimed to determine how sympathetic neuronal signalling and substrate stiffness jointly regulate VSMC behaviour under ageing-associated mechanical conditions by establishing an in vitro neurovascular co-culture model that incorporates both neural and mechanical cues relevant to the vascular microenvironment.

## 2. Materials and Methods

### 2.1. Isolation of Sympathetic Neurons and Source of VSMCs

Sympathetic neurons were isolated from the superior cervical ganglia (SCG) of neonatal Sprague–Dawley rats (P1–P3) following the protocol described by Zareen and Greene [32]. Newborn Sprague–Dawley rat pups (Charles River, Harlow, UK) were killed by cervical dislocation, followed by decapitation as a confirmation method by trained staff, in accordance with the guidelines of Imperial College’s Animal Welfare and Ethical Review Body (AWERB). Animals were maintained under standard laboratory housing conditions with controlled temperature and humidity, a 12 h light/dark cycle, ad libitum access to food and water, and appropriate environmental enrichment. Husbandry and animal care were provided in accordance with institutional standards, and all procedures followed the UK Animals (Scientific Procedures) Act 1986. In brief, rats were euthanised, and the SCGs were immediately transferred into ice-cold RPMI-1640 medium (Gibco, Thermo Fisher Scientific, Waltham, MA, USA; Cat. No.: 11875-093). The isolated ganglia were digested using 1 mL of 0.25% trypsin (Gibco, Thermo Fisher Scientific, USA; Cat. No.: 11560626) at 37 °C for 35 min. Trypsin was neutralised with the addition of 10 mL RPMI 1640 media containing 10% horse serum (Sigma-Aldrich, Merck, St. Louis, MO, USA; Cat. No.: H1138) and 5% foetal bovine serum (FBS; Sigma-Aldrich, USA; Cat. No.: F7524). The ganglia were then transferred into 1 mL of final media containing RPMI 1640 supplemented with 1% Pen/Strep, 1% horse serum, and 0.1% nerve growth factor. The ganglia were then dissociated by repeated pipetting (~20 times) using fire-polished glass Pasteur pipettes of decreasing diameters to obtain single neuronal cells in suspension.

The A7r5 rat VSMC cell line (ATCC, Manassas, VA, USA; Cat. No.: CRL-1444), derived from the thoracic aorta of embryonic rats, was used as the VSMC model. Cells were washed with phosphate-buffered saline (PBS; Sigma-Aldrich, UK; Cat. No.: P4417), digested with 1.5 mL of 0.25% trypsin at 37 °C for 8 min, and neutralised with 2.5 mL of complete culture medium to obtain a single-cell suspension. The complete culture medium consisted of Dulbecco’s Modified Eagle Medium (DMEM; Gibco, Thermo Fisher Scientific, USA; Cat. No.: 3042509) supplemented with 10% foetal bovine serum, 1% penicillin–streptomycin (Sigma-Aldrich, USA; Cat. No.: A5955), 0.2% sodium pyruvate (Sigma-Aldrich, USA; Cat. No.: S8636), 0.1% non-essential amino acids (NEAA; Sigma-Aldrich, USA; Cat. No.: M7145), and 0.1% insulin–transferrin–selenium (ITS; Sigma-Aldrich, USA; Cat. No.: I3146).

### 2.2. Fabrication of PDMS Substrates

PDMS-coated substrates were prepared to generate defined mechanical environments. Substrates with elastic moduli of 20 kPa and 130 kPa were used to represent the vascular mechanical conditions of healthy and aged arteries, respectively.

Sylgard 184 and Sylgard 527 silicone elastomers (Dow Corning, Midland, MI, USA) were mixed at mass ratios of 1:10 and 1:5 to obtain the desired stiffness values. The mixtures were thoroughly mixed, spin-coated onto 13 mm glass coverslips using a 150i spin processor (SPS) and cured overnight at 60 °C. Untreated 13-mm glass coverslips were included as a control condition.

### 2.3. Establishment of the VSMC–Sympathetic Neuron Co-Culture Model

Sympathetic neurons and VSMCs were initially seeded at a ratio of 20:1, as previously described [33]. However, to ensure sufficient neuronal contact during the 7-day culture period, the seeding density needed to be adjusted to 15:2 (30,000 VSMCs and 4000 neurons per well) per 13 mm glass coverslip.

Cells were seeded onto 13-mm coverslips under three substrate conditions: 20 kPa PDMS, 130 kPa PDMS, and glass. Prior to seeding, coverslips were coated with fibronectin (1:2000 in sterile PBS; Sigma-Aldrich, Gillingham, UK; Cat. No.: F1141) for 40 min at room temperature to promote cell adhesion.

Cells were maintained at 37 °C with 5% CO_2_ for 7 days in complete culture medium, and media were replaced every 1–2 days. To support neuronal survival and axonal extension, 100 ng/mL of fresh nerve growth factor (NGF; Sigma-Aldrich, UK; Cat. No.: SRP4304) was added to the culture media in both monocultures and cocultures.

### 2.4. Immunocytochemistry for Cell Area and γH2AX Detection

Cells were fixed with 4% paraformaldehyde (Thermo Fisher Scientific, USA; Cat. No.: J19943-K2) for 10 min at room temperature and washed three times with PBS. Cells were permeabilised and blocked in PBS containing 5% bovine serum albumin (BSA; Sigma-Aldrich, USA; Cat. No.: A7906-100G) and 0.1% Triton X-100 (Sigma-Aldrich, USA; Cat. No.: T9284) for 30 min at room temperature.

Primary antibodies were incubated overnight at 4 °C, including rabbit anti-γH2AX (Cell Signaling Technology, Danvers, MA, USA; Cat. No.: 9718S; 1:200) and chicken anti-tyrosine hydroxylase (TH) (Merck Millipore, Burlington, MA, USA; Cat. No.: AB9702; 1:100). After washing with PBS, cells were incubated with Alexa Fluor 488 donkey anti-rabbit (Invitrogen, Thermo Fisher Scientific, USA; Cat. No.: A11008; 1:500) and Alexa Fluor 647 goat anti-chicken secondary antibodies (Invitrogen, Thermo Fisher Scientific, USA; Cat. No.: A21436; 1:500) for 45 min at room temperature in the dark.

Alexa Fluor 568-phalloidin (Biotium, Fremont, CA, USA; Cat. No.: 00027-T; 1:400) and DAPI (Invitrogen, Thermo Fisher Scientific, USA; Cat. No.: 62249; 1:1000) were used to label F-actin and nuclei, respectively. Samples were mounted with ProLong™ Gold Antifade Reagent (Invitrogen, Thermo Fisher Scientific, USA; Cat. No.: P36930).

Images were acquired using a STELLARIS 8 inverted confocal microscope (Leica Microsystems, Wetzlar, Germany). Image analysis was performed using Fiji-ImageJ (v1.54d). The cell spreading area was quantified based on F-actin staining, and γH2AX signals were quantified relative to the nuclear area defined by DAPI staining.

### 2.5. EdUClick Assay for Proliferation Analysis

Cell proliferation was assessed using an EdUClick assay kit (Merck; Cat. No.: BCK-EDU-488) according to the manufacturer’s instructions. After 7 days of culture, the medium was replaced with fresh media containing 10 μM EdU, and cells were incubated at 37 °C for 1 h.

Cells were then washed with PBS containing 1% BSA and fixed using the kit fixative for 15 min at room temperature in the dark. Following fixation, cells were permeabilised using the kit permeabilisation reagent diluted 1:10 in 1% BSA-PBS for 15 min. The click reaction was performed using freshly prepared reaction solution and incubated for 30 min at room temperature in the dark.

Cells were subsequently stained with DAPI (1:1000) to label nuclei and mounted using ProLong™ Gold Antifade Reagent. Fluorescence images were acquired using a STELLARIS 8 inverted confocal microscope (Leica Microsystems, Germany). Image analysis was performed using Fiji ImageJ (v1.54d). EdU-positive cells and total nuclei were counted, and the ratio of EdU-positive cells to total cells was calculated as the proliferation rate.

### 2.6. Scratch Assay for Cell Migration

Cell migration was assessed using a scratch assay. After 7 days of culture, cells formed a confluent monolayer. A sterile 1000 µL pipette tip was used to generate a linear scratch across the monolayer, followed by washing with PBS to remove detached cells and the addition of fresh culture media.

Images of the scratch area were acquired at 0, 6, 24, and 48 h using an inverted fluorescence microscope (Axio Observer Z1, Zeiss, Jena, Germany). The wound area at each time point was quantified using Fiji ImageJ (v1.54d). Migration was expressed as the relative remaining scratch area (%), calculated as follows: Relative Remaining Scratch Area (%) = (Open Area T1/Open Area T0) × 100%.

### 2.7. Young’s Modulus (YM) Measurement

Young’s modulus (YM), indicative of cell stiffness, was measured using mechano-scanning ion conductance microscopy (SICM). SICM is a non-contact scanning probe technique enabling high-resolution topographical and mechanical measurements of live cells under physiological conditions, adapted from previously described pressure-based approaches [34,35]. For this work, no pressure was applied to the nanopipette; the transverse YM of the cells was measured by mechano-SICM using the intrinsic interaction between the nanopipette and the cell surface [36].

Briefly, a borosilicate glass nanopipette filled with electrolyte solution (144 mM NaCl, 10 mM HEPES, 1 mM MgCl_2_, and 5 mM KCl) was connected to an Ag/AgCl electrode and mounted on a *Z*-axis piezoelectric actuator. A reference electrode was placed in the bath solution to complete the electrochemical circuit. Changes in ionic current during probe approach were used for feedback control to achieve non-contact surface scanning.

Measurements were performed using hopping probe ion conductance microscopy mode, which allows point-by-point scanning from a reference height to minimise surface disturbance. The transverse YM was determined by recording the *Z*-axis displacement (Δd) corresponding to 1% and 2% reductions in ionic current (SP1 and SP2). The transverse YM was calculated using the relationship YM = I/Δd, where I represents the estimated intrinsic stress.

Image analysis was performed using SICM Image Viewer software 1.2.5.10 (ICAPPIC Ltd., London, UK) with quantitative nanomechanical mapping (QNM).

### 2.8. Statistical Analysis

All data presented in this work were assessed with Shapiro-Wilk test and found to be not normally distributed. For comparisons between two groups, the Mann–Whitney test was used. For multiple-group comparisons, the Kruskal–Wallis test followed by Dunn’s multiple-comparison test was applied. A *p* value < 0.05 was considered statistically significant.

## 3. Results

### 3.1. Establishment of the Co-Culture System

VSMC monoculture (SMC) or VSMC with sympathetic neuron co-culture (SMC_N) was plated on 13 mm glass coverslips. After 7 days of culture, cells were examined using an inverted fluorescence microscope.

**Figure 1:**
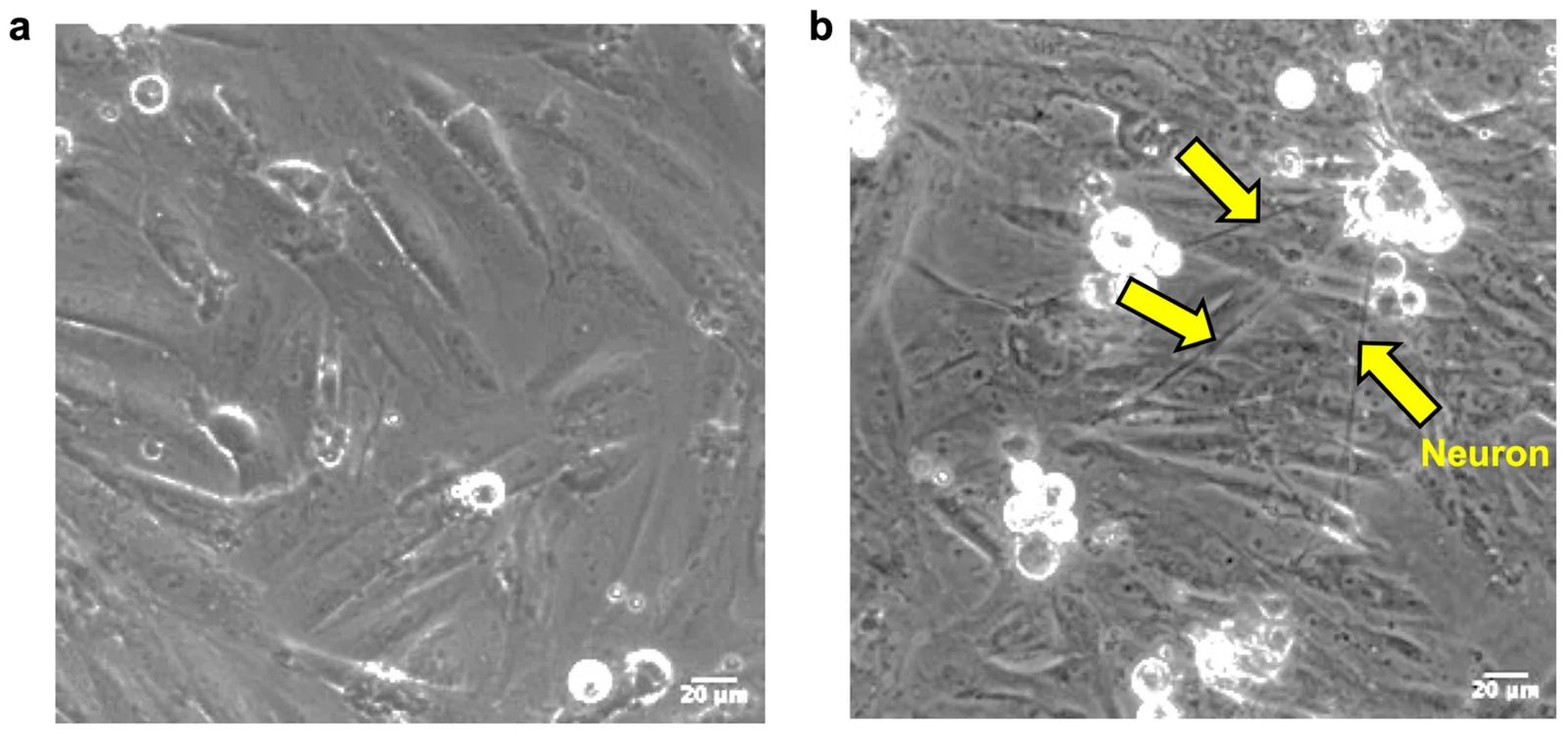
Establishment of the VSMC–sympathetic neuron co-culture system. Brightfield images of VSMCs cultured alone (SMC) or together with sympathetic neurons (SMC_N) on glass coverslips after 7 days of culture: (**a**) VSMC monoculture showing the typical spindle-shaped morphology of VSMCs. (**b**) In co-culture, neuronal processes extend toward and contact VSMCs (yellow arrows). Images were acquired using an inverted fluorescence microscope (20× objective). Scale bar = 20 μm.

In monoculture, VSMCs displayed a typical spindle-shaped morphology and were evenly distributed across the substrate (Figure 1a). In the co-culture condition, neurite extensions from sympathetic neurons were observed contacting VSMCs (yellow arrows, Figure 1b), indicating successful establishment of neuron–VSMC interactions in the co-culture condition.

### 3.2. Co-Culture Reduces VSMC Spreading and Is Modulated by Substrate Stiffness

**Figure 2:**
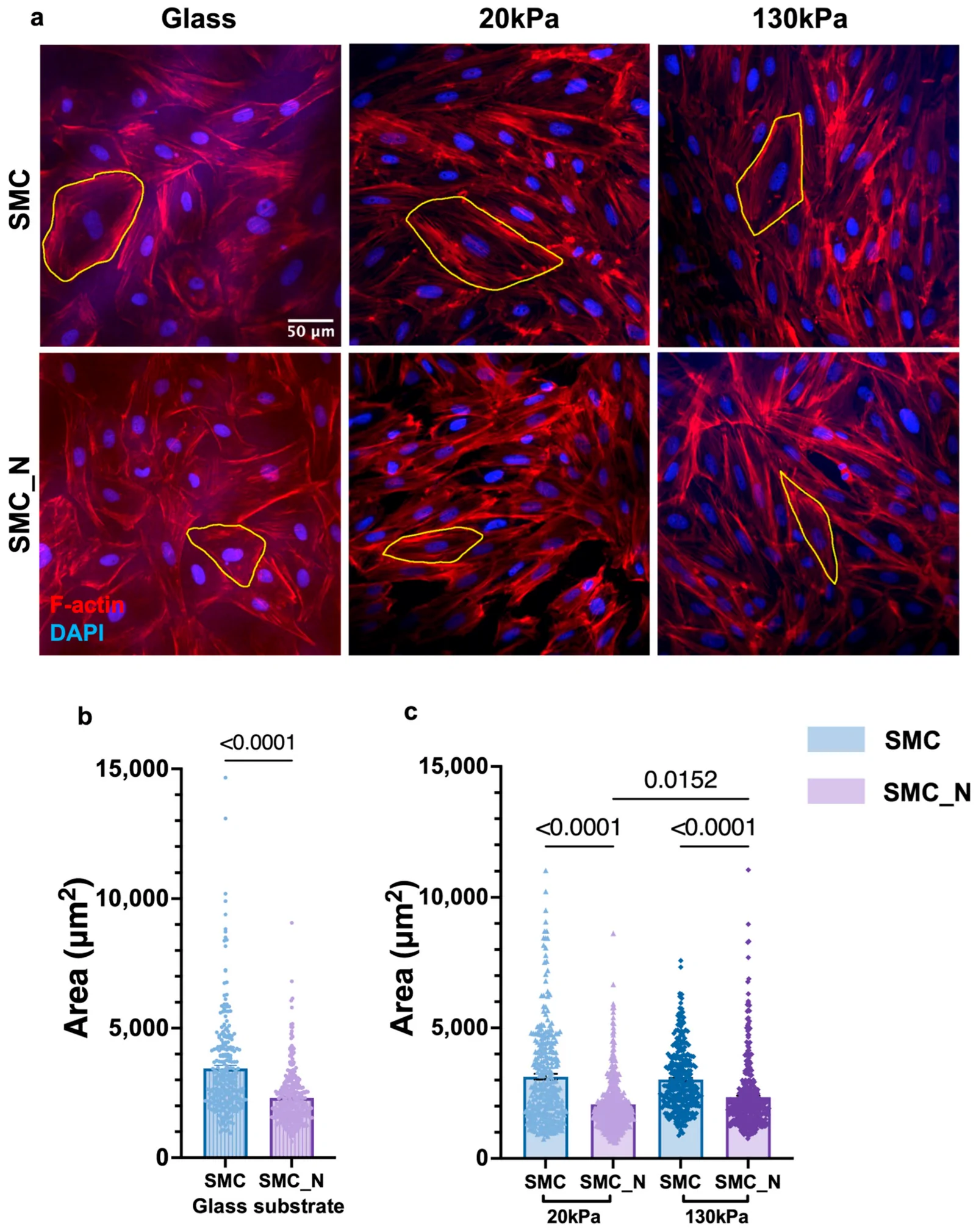
Effects of sympathetic neuron co-culture and substrate stiffness on VSMC area: (**a**) Representative immunofluorescence images of VSMCs cultured alone (SMC) or with sympathetic neurons (SMC_N) on glass, 20 kPa PDMS, and 130 kPa PDMS substrates. F-actin (red) and nuclei (DAPI, blue) are shown. Yellow outlines indicate representative cells used for area measurements. Scale bar = 50 μm. (**b**) Quantification of VSMC area on glass substrates comparing SMC and SMC_N conditions. (**c**) Quantification of VSMC area on PDMS substrates with different stiffness (20 kPa and 130 kPa) in monoculture and co-culture conditions. Data are presented as mean ± SEM. Statistical analysis was performed using the Mann–Whitney test for two-group comparisons and the Kruskal–Wallis test followed by Dunn’s multiple-comparison test for multiple-group comparisons. *N* = 3 independent experiments; *n* = 263–363 for (**b**); *n* = 299–408 for (**c**); n indicates the number of analysed cells for each condition.

The cell spreading area reflects the VSMC phenotypic state and serves as a functional readout of morphological responses to the mechanical and chemical microenvironment. To assess how sympathetic neuron co-culture and substrate stiffness jointly influence VSMC morphology, cell area was quantified by F-actin staining after 7 days of culture. Representative images of SMC monoculture and SMC_N co-cultured on glass and PDMS substrates are shown in Figure 2a.

On glass substrates, VSMC area was statistically significantly reduced under SMC_N co-culture conditions compared with SMC monoculture (*p* < 0.0001, Figure 2b), indicating that the presence of sympathetic neurons was associated with reduced VSMC spreading.

To examine whether substrate stiffness influences neuronal regulation of VSMC morphology, cells were cultured on PDMS substrates with elastic moduli of 20 kPa (mimicking healthy arteries) and 130 kPa (mimicking aged arteries).

Consistent with observations on glass substrates, VSMC area was also reduced under SMC_N co-culture compared with SMC monoculture at both stiffness levels (*p* < 0.0001, Figure 2c). However, this reduction was lower on the 130 kPa substrate than on the 20 kPa substrate, indicated by a greater VSMC area in co-culture on 130 kPa substrates compared with 20 kPa substrates (*p* = 0.0152, Figure 2c). In monoculture, no significant difference in VSMC area was observed between PDMS substrate conditions (Figure 2c).

### 3.3. Effects of Neuronal Co-Culture and Substrate Stiffness on VSMC Proliferation

**Figure 3:**
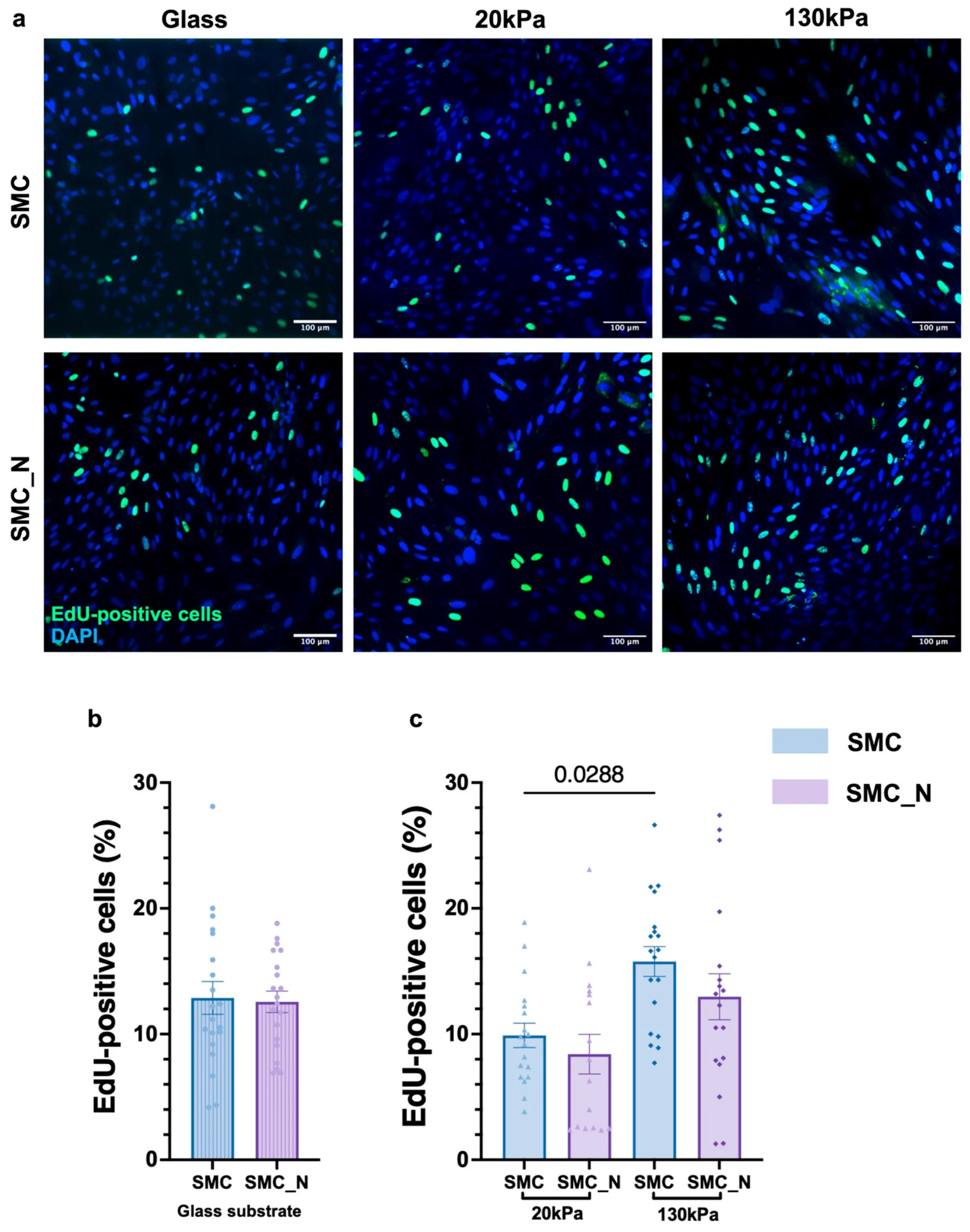
Effects of sympathetic neuron co-culture and substrate stiffness on VSMC proliferation: (**a**) Representative immunofluorescence images of EdU incorporation in SMC and SMC_N cultures. EdU-positive nuclei are shown in green, and total nuclei are labelled with DAPI (blue). Scale bar = 100 μm. (**b**) Quantification of the proportion of EdU-positive cells in SMC and SMC_N cultures on glass substrates. (**c**) Quantification of EdU-positive cells on PDMS substrates with elastic moduli of 20 kPa and 130 kPa. Data are presented as mean ± SEM. Statistical analysis was performed using the Mann–Whitney test for two-group comparisons and the Kruskal–Wallis test followed by Dunn’s multiple-comparison post hoc test for multi-group comparisons. *N* = 3 independent experiments; *n* = 20 for (**b**); *n* = 16–18 for (**c**).

VSMC proliferation is a key driver of vascular remodelling and disease progression. To examine whether neuronal co-culture and substrate stiffness modulate this response, proliferation was assessed using an EdU incorporation assay after 7 days of culture. Representative images of EdU staining in SMC and SMC_N groups are shown in Figure 3a.

On glass substrates, EdU-positive cells were detected in both monoculture and co-culture conditions. Quantitative analysis showed that the proportion of EdU-positive cells did not differ statistically between the SMC and SMC_N groups (Figure 3b).

To examine whether substrate mechanics influence proliferative responses, cells were cultured on PDMS substrates with elastic moduli of 20 kPa and 130 kPa. In monoculture, the proportion of EdU-positive cells was statistically significantly higher on the 130 kPa substrate compared with the 20 kPa substrate (*p* = 0.0288, Figure 3c). In contrast, no statistical difference between stiffness conditions was observed in SMC_N cultures (Figure 3c).

### 3.4. Effects of Neuronal Co-Culture on VSMC Migration

**Figure 4:**
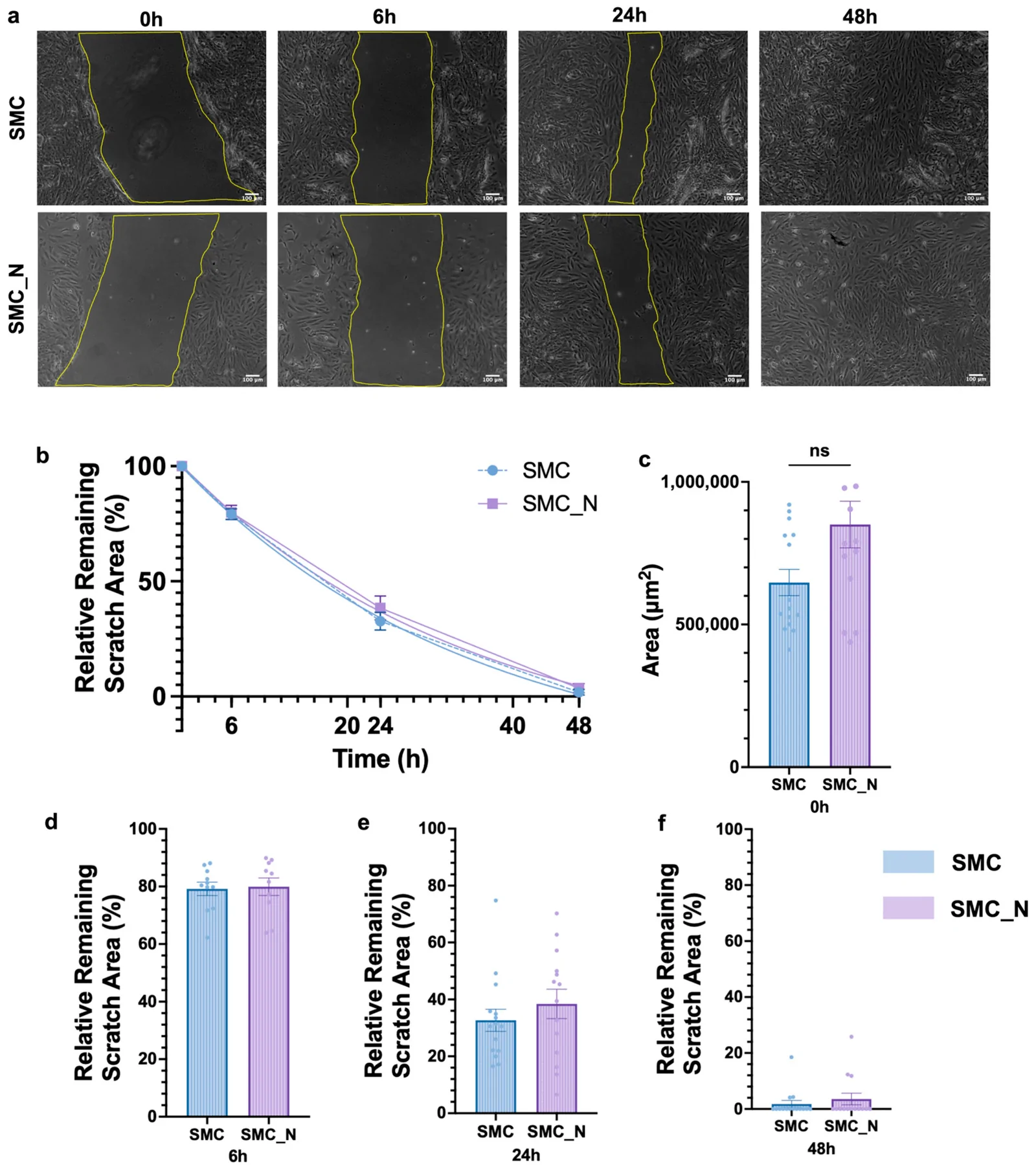
Effects of neuronal co-culture on VSMC migration: (**a**) Representative wound healing images of SMC and SMC_N cultured on glass surfaces at 0, 6, 24, and 48 h. Scale bar = 100 μm. (**b**) Nonlinear curve fitting of wound closure using a one-phase decay model: SMC, Y(t) = 126.01·e^−0.03181·t^ − 25.71; SMC_N, Y(t) = 139.66·e^−0.02421·t^ − 40.01. No statistically significant difference was observed (*p* = 0.3801). (**c**) Comparison of initial wound area (0 h) revealed no statistical difference (*N* = 3, *n* = 14–15). (**d**–**f**) Percentage of remaining scratch area at 6, 24, and 48 h. Data are presented as mean ± SEM (*N* = 3, *n* = 10–15). Statistical analysis was performed using the Mann–Whitney U test.

VSMC migration is a critical process in vascular remodelling, yet whether sympathetic neuronal signalling modulates this behaviour remains unclear. To investigate this, migration was evaluated using a scratch wound assay on glass substrates. Representative images of wound closure in SMC and SMC_N cultures on glass substrates are shown in Figure 4a.

The relative remaining scratch area was quantified over time and fitted using a one-phase decay model (Figure 4b). Comparison of the fitted curves revealed no statistical difference between SMC and SMC_N conditions (extra sum-of-squares F test, *p* = 0.3801). Consistently, analysis of the relative wound area at individual time points showed no statistical differences between groups (Figure 4c).

To explore whether substrate mechanics influence migration, scratch assays were also performed on PDMS substrates with elastic moduli of 20 kPa and 130 kPa. However, mechanical disruption of the flexible PDMS surface during scratching frequently produced irregular wound edges and surface damage within the wound region. Although limited cell movement toward the wound centre was observed, reliable wound closure measurements could not be obtained, preventing quantitative analysis on PDMS substrates.

### 3.5. Effects of Neuronal Co-Culture and Substrate Stiffness on VSMC Young’s Modulus

**Figure 5:**
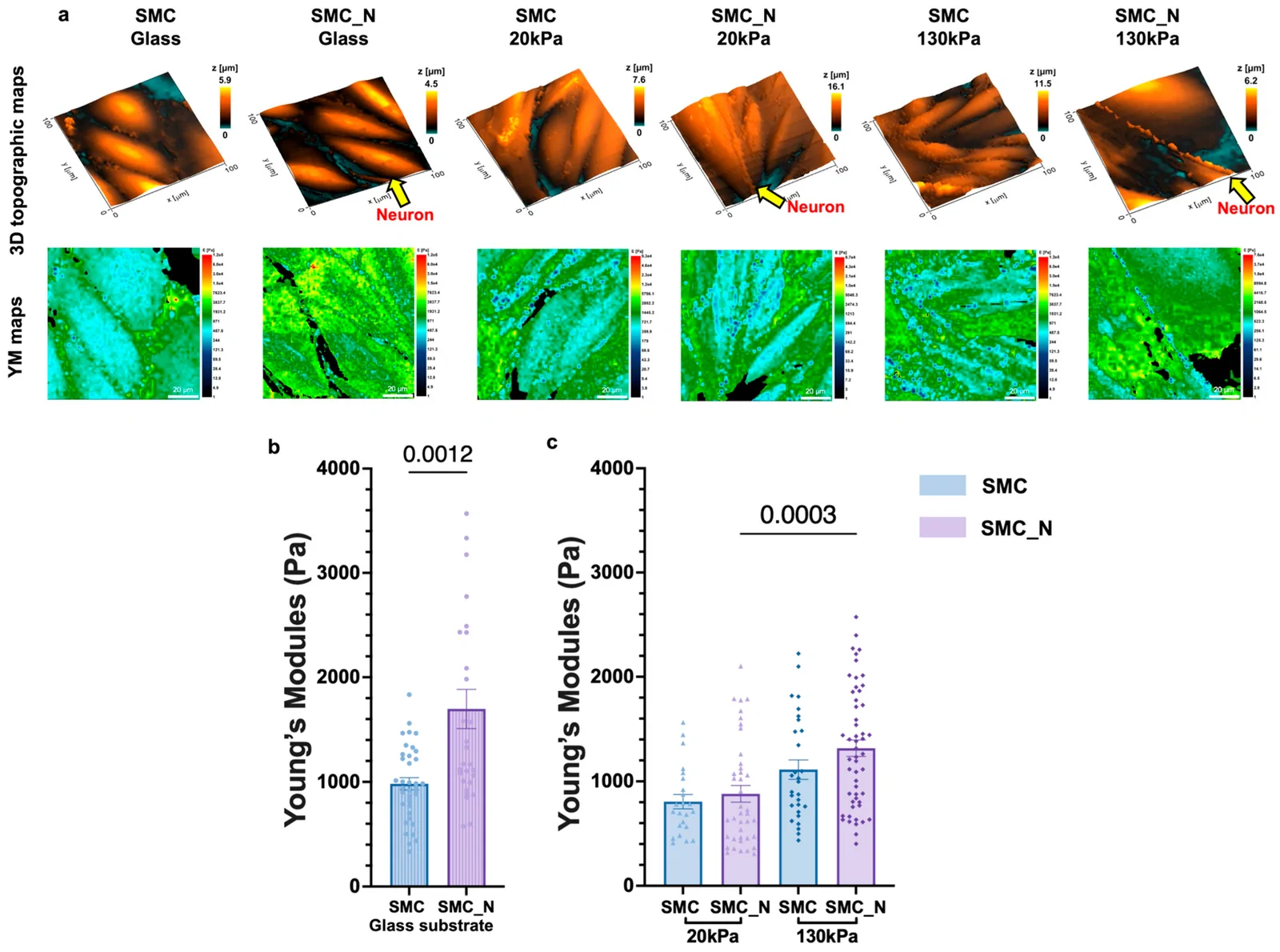
Effects of neuronal co-culture and substrate stiffness on VSMC Young’s modulus measured by SICM: (**a**) Representative SICM topography and YM maps of VSMCs cultured under monoculture (SMC) and co-culture (SMC_N) conditions. (**b**) Quantification of YM measured by SICM showing increased YM in SMC_N compared with SMC cultures on glass substrates. (**c**) Quantification of VSMC YM on PDMS substrates with elastic moduli of 20 kPa and 130 kPa under SMC and SMC_N conditions. Data are presented as mean ± SEM. Statistical analysis was performed using the Mann–Whitney test for two-group comparisons and the Kruskal–Wallis test followed by Dunn’s multiple-comparison post hoc test for multi-group comparisons. *N* = 3 independent experiments; n = 29–37 for (**b**); *n* = 23–53 for (**c**).

Cell YM reflects VSMC cytoskeletal organisation and is altered under pathological conditions, including ageing-associated vascular stiffening. To assess whether neuronal co-culture and substrate stiffness further elevate or attenuate this response, VSMC YM was measured using Mechano-Scanning Ion Conductance Microscopy (mechano-SICM). Representative SICM images and YM maps are shown in Figure 5a.

Quantitative analysis demonstrated that VSMC YM was statistically higher in SMC_N co-cultures compared with SMC monocultures. The average YM increased from 982.4 Pa in SMC cells to 1698 Pa in SMC_N cells (*p* = 0.0012, Figure 5b).

To examine whether substrate mechanics influence cellular YM, VSMCs were cultured on PDMS substrates with elastic moduli of 20 kPa and 130 kPa. Consistent with observations on glass substrates, a tendency towards increased YM was observed under SMC_N co-culture conditions; however, the differences were not statistically significant between the SMC and SMC_N groups under either PDMS condition. Across both culture conditions, cells cultured on the 130 kPa substrate consistently exhibited statistically higher YM than those on the 20 kPa substrate (*p* = 0.0003, Figure 5c).

### 3.6. Effects of Neuronal Co-Culture and Substrate Stiffness on VSMC DNA Damage

**Figure 6:**
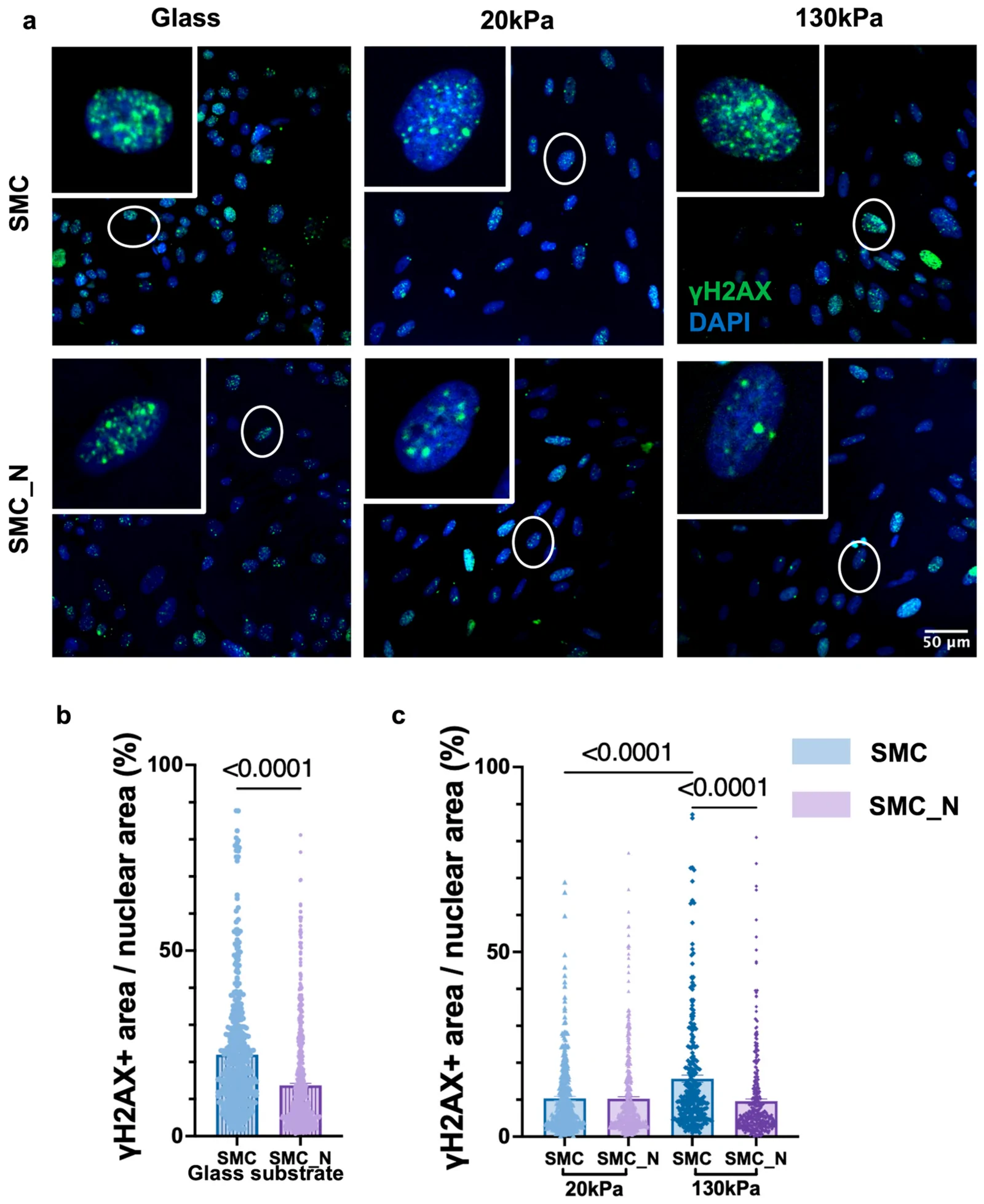
Effects of neuronal co-culture and substrate stiffness on VSMC DNA damage: (**a**) Representative immunofluorescence images showing γH2AX staining (green) and DAPI-labelled nuclei (blue) in SMC and SMC_N cultures on glass, 20 kPa, and 130 kPa substrates. Scale bar = 50 μm. (**b**) Quantification of γH2AX-positive nuclear area ratio in SMC and SMC_N cultures on glass substrates. (**c**) Quantification of γH2AX levels in SMC and SMC_N cultures on PDMS substrates with elastic moduli of 20 kPa and 130 kPa. Data are presented as mean ± SEM. Statistical analysis was performed using the Mann–Whitney test for two-group comparisons and the Kruskal–Wallis test followed by Dunn’s multiple-comparison post hoc test for multi-group comparisons. *N* = 3 independent experiments; *n* = 454–641 for (**b**); *n* = 330–453 for (**c**).

This study assessed γH2AX expression to investigate the roles of substrate stiffness and sympathetic innervation in VSMC DNA damage. Representative staining images of SMC and SMC_N cultures are shown in Figure 6a.

On glass substrates, quantitative analysis showed that the γH2AX-positive nuclear area ratio was statistically lower in SMC_N cultures compared with SMC monocultures (*p* < 0.0001, Figure 6b).

To examine whether substrate mechanics influence DNA damage responses, cells were cultured on PDMS substrates with elastic moduli of 20 kPa and 130 kPa. In monoculture, γH2AX levels were statistically higher on the 130 kPa substrate than on the 20 kPa substrate (*p* < 0.0001). In contrast, no significant difference between stiffness conditions was observed in SMC_N cultures. When both factors were analysed together, γH2AX levels at 130 kPa were statistically lower in SMC_N co-cultures than in SMC monocultures, whereas no difference was detected between groups at 20 kPa (Figure 6c).

## 4. Discussion

**Figure 7:**
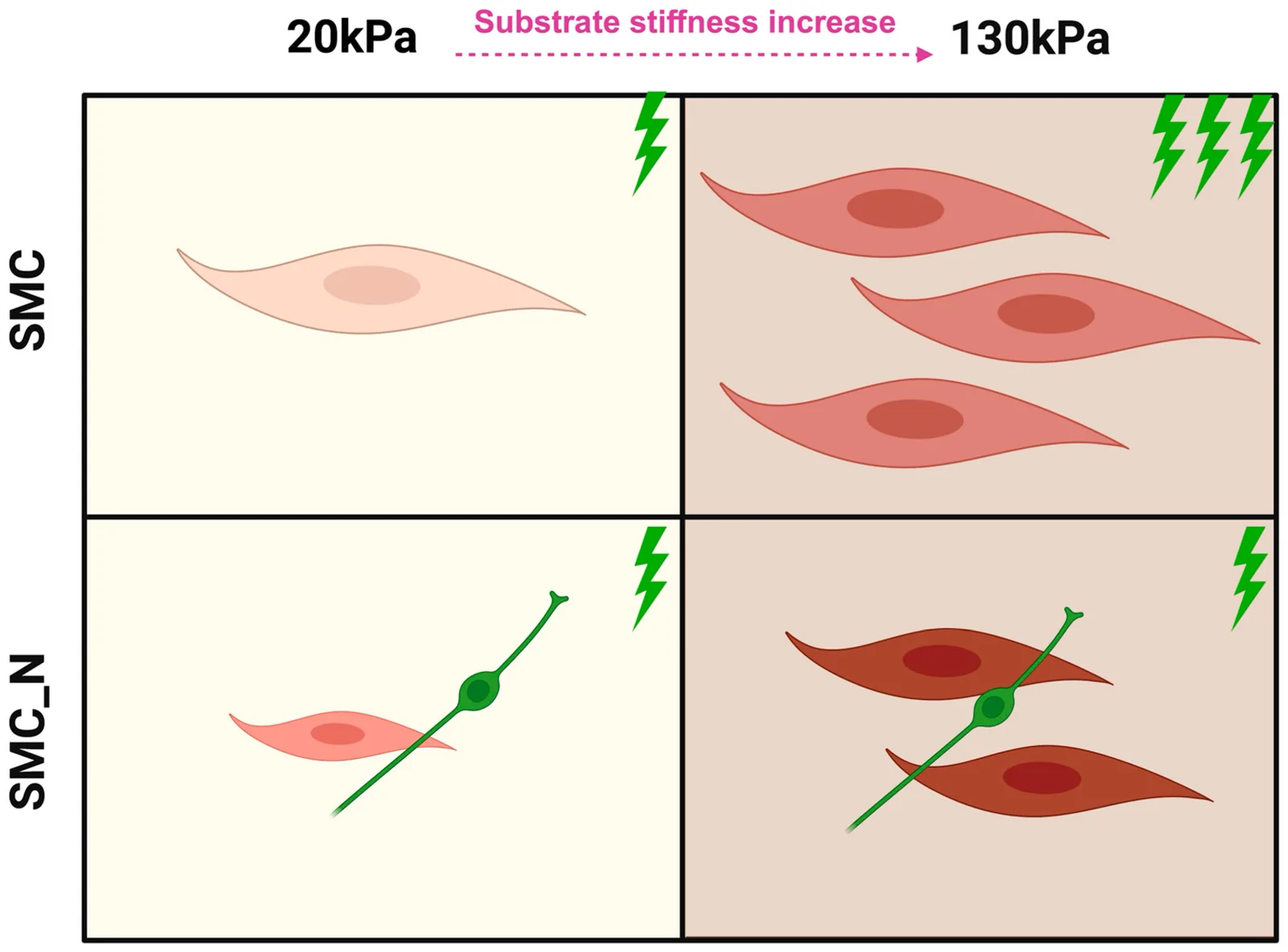
Schematic summary model. Cell size indicates the extent of surface spreading; the number of cells reflects proliferation, with higher counts denoting increased proliferation rates. Cell colour intensity, ranging from light pink to dark red, represents progressive increases in cellular YM. Green lightning symbols denote γH2AX, a marker of ageing-associated DNA damage, with greater numbers indicating more severe damage.

Sympathetic neuronal co-culture was associated with reduced VSMC spreading and decreased γH2AX levels. Under the conditions tested, neural signalling exerted limited effects on cell proliferation and migration. In contrast, increased substrate stiffness influenced cell proliferation and mechanical properties. Both neuronal input and substrate stiffness were associated with increased VSMC YM (Figure 7). This framework provides a controllable platform for investigating neuro-mechanical regulation in vascular smooth muscle biology and supports the development of more physiologically relevant neurovascular in vitro models.

A major limitation of conventional in vitro VSMC studies is the widespread use of glass or plastic culture surfaces with elastic moduli far exceeding the physiological range of arterial tissue, which can drive abnormal spreading and promote a synthetic-like phenotype [37,38]. Tuneable materials such as polydimethylsiloxane (PDMS) provide an experimentally tractable platform to mimic defined stiffness conditions and have been widely adopted to investigate how mechanical cues regulate cell behaviour [39]. Nonetheless, PDMS-based systems remain simplified representations of the in vivo niche, highlighting the importance of careful interpretation and the value of mechanistic, variable-controlled in vitro studies.

In this study, glass substrates were retained as a conventional reference condition to enable direct comparison with previous literature. The inclusion of both glass and tuneable PDMS substrates allowed us to distinguish between responses observed under standard in vitro conditions and those occurring within more physiologically relevant mechanical environments.

### 4.1. Neuronal Modulation of VSMC Morphology Under Ageing-Associated Mechanical Conditions

Cell area is closely associated with VSMC phenotype and cytoskeletal organisation and is commonly used as an indicator of morphological state [40]. In the present study, sympathetic neuron co-culture was associated with a reduced VSMC spreading area, suggesting that neuronal signalling may influence cell morphology by limiting cell expansion (Figure 2).

Sympathetic neurons release neurotransmitters that regulate smooth muscle phenotype and cytoskeletal dynamics [23]. Previous studies have shown that factors such as norepinephrine (NE) can enhance the expression of contractile markers, including α-SMA and smoothelin, and activate signalling pathways such as RhoA/ROCK, which contribute to cytoskeletal tension and morphological regulation [23]. Smooth muscle cells also express functional adrenergic receptors, and stimulation with agonists such as NE or phenylephrine can elevate intracellular calcium and induce contractile responses [41]. However, neural regulation is context-dependent. Excessive sympathetic signalling has been reported to promote VSMC dedifferentiation and increased cell spreading in abdominal aortic aneurysm models [42].

Mechanical cues also play an important role in determining VSMC morphology. Substrate stiffness can regulate cytoskeletal organisation through integrin-mediated adhesion and RhoA/ROCK signalling, influencing the balance between contractile and synthetic phenotypes [43]. Consistent with this, increased substrate stiffness in the present study was associated with greater VSMC spreading. Notably, neuronal modulation of VSMC area was observed across different substrate stiffness conditions, although the effect appeared less pronounced at higher stiffness (130 kPa), suggesting that mechanical cues associated with ageing-related vascular stiffening may influence the extent of neuronal regulation.

### 4.2. Substrate Stiffness Predominates in Regulating VSMC Proliferation Under Ageing-Associated Mechanical Conditions

VSMC proliferation is a key process in vascular remodelling and contributes to pathological changes such as intimal hyperplasia and luminal narrowing [44]. In the present study, sympathetic neuron co-culture did not significantly alter VSMC proliferation, whereas increased substrate stiffness (130 kPa) was associated with a higher proportion of EdU-positive cells (Figure 3). These findings suggest that substrate mechanical properties represent an important determinant of VSMC proliferation.

Previous studies have shown that sympathetic neurotransmitters can regulate VSMC proliferation in a context-dependent manner. For example, NE has been reported to stimulate proliferation through activation of α1-adrenergic receptors, whereas under other conditions it may suppress abnormal proliferation through inhibition of TGF-β signalling [45,46,47]. These findings indicate that the proliferative response of VSMCs to neural input depends strongly on transmitter concentration and microenvironmental context. In the present co-culture model, neurotransmitters were released endogenously by sympathetic neurons rather than applied at defined concentrations. Under these conditions, neural signalling may remain within a physiological range and therefore may not produce a strong proliferative response. Similar observations have been reported in the PC12–A10 co-culture system, where neuronal signalling primarily promoted contractile characteristics rather than increased proliferation [23].

In contrast, the influence of substrate stiffness appeared more pronounced. Increased matrix stiffness, commonly associated with vascular ageing, has been linked to enhanced VSMC proliferative activity in several experimental models [48,49,50]. Consistent with these observations, our findings indicate that substrate stiffness represents a dominant factor regulating VSMC proliferation within the ageing-associated mechanical environment examined in this study.

### 4.3. Neural Signalling and Substrate Stiffness in VSMC Migration

VSMC migration is an important process in vascular remodelling and disease progression [51]. In the present study, scratch assays showed that sympathetic neuron co-culture did not significantly alter the overall migration rate of VSMCs under the experimental conditions used (Figure 4). Previous studies have suggested that sympathetic neurotransmitters such as NE can influence VSMC phenotype by enhancing contractile marker expression and limiting the transition toward a synthetic phenotype, which is typically associated with increased migratory activity [23]. These observations indicate that neural signalling may contribute to maintaining a contractile cellular state, potentially restricting migration.

In the present study, migration experiments on PDMS substrates were technically limited because scratching produced irregular surfaces that interfered with reliable quantification. As a result, the present study is unable to directly determine whether or how substrate stiffness influences VSMC migration, and this aspect of neuro-mechanical regulation could only be assessed on glass substrates. The relationship between matrix stiffness and VSMC migration described below is therefore based on previous literature rather than direct experimental evidence from this study. Previous work has reported that increased substrate stiffness enhances VSMC responsiveness to growth factors such as PDGF and promotes migratory behaviour [48] and that VSMC migration exhibits a biphasic relationship with substrate stiffness, with maximal migration occurring within an intermediate stiffness range [52]. Whether a similar relationship applies to the present neuro-mechanical co-culture system remains to be established.

### 4.4. Neuronal Influences on VSMC YM Under Ageing-Associated Mechanical Conditions

VSMC YM is an important contributor to vascular mechanical properties and functional homeostasis [51]. Although arterial stiffening has traditionally been attributed to extracellular matrix (ECM) remodelling, increasing evidence suggests that intrinsic cellular YM of VSMCs also plays a role in determining vascular mechanics [53]. In the present study, VSMC YM was statistically higher in the sympathetic neuron co-culture group than in monoculture, suggesting that neuronal input can influence cellular mechanical properties (Figure 5).

One possible explanation is that neurotransmitters such as NE activate adrenergic receptors on VSMCs and enhance cytoskeletal contractility through calcium-dependent signalling and actin–myosin interactions. Previous physiological studies have shown that sympathetic activation can increase arterial stiffness or vascular tone [54,55]. Although these studies mainly assessed vascular function at the tissue level, they support the possibility that neural signalling may influence arterial mechanics through modulation of VSMC mechanical behaviour.

Substrate mechanics also appeared to contribute to VSMC YM. In this study, cells cultured on the stiffer substrate (130 kPa) exhibited increased stiffness regardless of neuronal co-culture. Increased matrix stiffness is commonly associated with ageing-related vascular stiffening and has been linked to enhanced cytoskeletal tension and stress fibre formation in VSMCs [53,56,57,58]. Consistent with these observations, our results suggest that both substrate stiffness and neural input may contribute to regulating VSMC mechanical properties within ageing-associated mechanical environments. However, the relative contribution of these factors and the underlying molecular mechanisms require further investigation.

### 4.5. Neuronal Modulation of DNA Damage in VSMCs Under Ageing-Associated Mechanical Conditions

γH2AX is a well-established marker of DNA double-strand breaks and an indicator of activation of the DNA damage response (DDR) [59]. In this study, γH2AX staining was used to evaluate DNA damage in VSMCs under different mechanical and neural conditions. The results showed that, in monoculture, higher substrate stiffness was associated with increased nuclear γH2AX levels, whereas this increase was attenuated under sympathetic neuronal co-culture, suggesting that neuronal input may partially modulate stiffness-associated DNA damage responses.

Substrate stiffening is an important feature of vascular ageing and has been associated with increased cytoskeletal tension, altered nuclear mechanical properties, and activation of mechanotransduction pathways [60,61]. Excessive mechanical stress may promote nuclear deformation and genomic instability through alterations in chromatin organisation and the transmission of cytoskeletal forces to the nucleus. The present findings support this notion, indicating that a stiffer substrate environment may increase DNA damage responses in VSMCs.

The reduction in γH2AX levels under neuronal co-culture suggests that sympathetic signalling may confer a degree of protection against stiffness-induced DNA damage. One possible explanation is that sympathetic neuron-derived factors can simultaneously regulate cellular stress responses and mechanical homeostasis. Previous studies have shown that norepinephrine (NE) can influence stress response pathways related to genomic stability, including PI3K–AKT and ATM signalling [62]. In addition, neuropeptide Y (NPY) has been reported to exert cytoprotective effects, such as reducing DNA damage and improving nuclear integrity [63]. Beyond these biochemical signalling effects, one possible mechanism is that neuronal input may also influence cytoskeletal organisation and cellular contractile state, thereby altering the transmission of mechanical forces to the nucleus. As nuclear mechanical stress is closely linked to mechanotransduction-mediated DNA damage, sympathetic signalling may attenuate stiffness-induced DNA damage by reducing nuclear mechanical loading.

Overall, the present study supports a working model in which ageing-associated mechanical stress promotes VSMC DNA damage responses, while sympathetic input may partially buffer this process by modulating cellular stress pathways and cytoskeleton–nucleus mechanical coupling. However, the specific molecular mechanisms mediating this neuroprotective effect require further investigation. Compared with conventional culture systems that focus on a single mechanical stimulus, the model established here enables investigation of interactions among multiple microenvironmental factors, offering a novel controllable platform for studying the synergistic effects of neuronal signalling and substrate stiffness on vascular ageing, and may inform the development of more physiologically relevant systems, such as organoid or organ-on-chip models. Although the specific molecular mechanisms underlying neural regulation of DNA damage and VSMC behavioural changes remain unresolved, the phenomena observed and the model established here provide an experimental basis for future work to elucidate neuro-mechanical crosstalk. From a physiological perspective, VSMC adaptive changes during vascular ageing may not be driven by ECM stiffening alone; incorporating neural regulation into mechanobiological research may help provide a more complete understanding of vascular remodelling. The present findings further suggest that neuronal signalling may participate in regulating mechanically induced DNA damage responses, offering a new perspective on the relationship between mechanical stimuli, neural regulation, and genomic stability during vascular ageing. Characterising the potential protective role of neural regulation in vascular homeostasis in greater detail may help identify novel intervention targets and inform the development of therapeutic strategies for vascular ageing-related disease.

## 5. Limitations

First, this study used the A7r5 rat aortic VSMC line rather than primary VSMCs. This choice was based on its culture stability, high reproducibility, and suitability for long-term culture and complex mechanical stimulation experiments, which have led to its widespread use in vascular smooth muscle cell research [64,65]. However, as an embryonically derived immortalised cell line, its phenotype, cytoskeletal organisation, and phenotypic plasticity may not fully reflect those of adult primary VSMCs, and extrapolation of the present findings to the in vivo vascular ageing context should therefore be made with caution [66]. Second, the PDMS-based in vitro model represents a simplified two-dimensional mechanical environment and does not fully recapitulate the three-dimensional vascular microenvironment, including complex ECM composition and multicellular interactions. Third, neuronal density, VSMC maturation state, and neurotransmitter release were not directly quantified in the co-culture system, which limits the interpretation of neural regulatory mechanisms. Therefore, although this model provides a controllable and reproducible experimental platform for studying neuro-mechanical interactions, the effects observed in this study warrant further validation in primary VSMCs or other more physiologically relevant models, in combination with in vivo studies, to confirm whether they similarly apply to the actual vascular environment.

## 6. Conclusions

This study established an in vitro sympathetic neuron–VSMC co-culture model to investigate how sympathetic innervation interacts with substrate mechanics under ageing-associated mechanical conditions. Sympathetic neuronal co-culture was associated with reduced VSMC spreading and decreased γH2AX levels, suggesting a potential protective role of neural signalling against ageing-related cellular stress. Under the conditions tested, neural signalling exerted limited effects on cell proliferation and migration. In contrast, increased substrate stiffness, mimicking the stiffer mechanical environment of aged arteries, drove elevated cell proliferation, increased cellular YM, and exacerbated DNA damage, which are hallmarks of ageing-associated vascular dysfunction. Both neuronal input and substrate stiffness were associated with increased cellular YM, indicating that the ageing mechanical microenvironment and sympathetic innervation may act in concert to alter VSMC biomechanical properties. Together, these findings suggest that neural and mechanical cues jointly shape VSMC behaviour within ageing-relevant mechanical environments. By integrating sympathetic neuronal signalling with the substrate mechanical environment, this study establishes a novel experimental framework for investigating neuro-mechanical regulation during vascular ageing and provides a basis for future exploration of the role of neural factors in vascular dysfunction and ageing-associated cardiovascular disease.

## Data Availability

The original contributions presented in this study are included in the article. Further inquiries can be directed to the corresponding authors.

## Abbreviations

The following abbreviations are used in this manuscript:

CVDs	Cardiovascular diseases
ECM	Extracellular matrix
NE	Norepinephrine
NGF	Nerve growth factor
PDMS	Polydimethylsiloxane
ROIs	Regions of interest
SICM	Scanning ion conductance microscopy
SMC	Smooth muscle cell
SMC_N	Smooth muscle cell and neuron
VSMC	Vascular smooth muscle cell
YM	Young’s modulus
